# Assessment of a one-week ketogenic diet on brain glycolytic metabolism and on the status epilepticus stage of a lithium–pilocarpine rat model

**DOI:** 10.1038/s41598-024-53824-4

**Published:** 2024-03-01

**Authors:** Matthieu Doyen, Clémentine Lambert, Emilie Roeder, Henri Boutley, Bailiang Chen, Julien Pierson, Antoine Verger, Emmanuel Raffo, Gilles Karcher, Pierre-Yves Marie, Fatiha Maskali

**Affiliations:** 1 NANCYCLOTEP-Molecular and Experimental Imaging Platform, 54000 Nancy, France; 2 Lorraine University, IADI, INSERM UMR 1254, 54000 Nancy, France; 3 Department of Neuropediatrics, Children’s Hospital CHRU Nancy, 54000 Nancy, France; 4 CHRU-Nancy, INSERM UMR 1433, CIC, Innovation Technologique, Université de Lorraine, 54000 Nancy, France; 5 Department of Nuclear Medicine, University Hospital, 54000 Nancy, France; 6 Lorraine University, INSERM DCAC1116, 54000 Nancy, France

**Keywords:** Epilepsy, Preclinical research, Molecular imaging

## Abstract

The ketogenic diet (KD) has been shown to be effective in refractory epilepsy after long-term administration. However, its interference with short-term brain metabolism and its involvement in the early process leading to epilepsy remain poorly understood. This study aimed to assess the effect of a short-term ketogenic diet on cerebral glucose metabolic changes, before and after status epilepticus (SE) in rats, by using [^18^F]-FDG PET. Thirty-nine rats were subjected to a one-week KD (KD-rats, n = 24) or to a standard diet (SD-rats, n = 15) before the induction of a status epilepticus (SE) by lithium-pilocarpine administrations. Brain [^18^F]-FDG PET scans were performed before and 4 h after this induction. Morphological MRIs were acquired and used to spatially normalize the PET images which were then analyzed voxel-wisely using a statistical parametric-based method. Twenty-six rats were analyzed (KD-rats, n = 15; SD-rats, n = 11). The 7 days of the KD were associated with significant increases in the plasma β-hydroxybutyrate level, but with an unchanged glycemia. The PET images, recorded after the KD and before SE induction, showed an increased metabolism within sites involved in the appetitive behaviors: hypothalamic areas and periaqueductal gray, whereas no area of decreased metabolism was observed. At the 4th hour following the SE induction, large metabolism increases were observed in the KD- and SD-rats in areas known to be involved in the epileptogenesis process late—i.e., the hippocampus, parahippocampic, thalamic and hypothalamic areas, the periaqueductal gray, and the limbic structures (and in the motor cortex for the KD-rats only). However, no statistically significant difference was observed when comparing SD and KD groups at the 4th hour following the SE induction. A one-week ketogenic diet does not prevent the status epilepticus (SE) and associated metabolic brain abnormalities in the lithium-pilocarpine rat model. Further explorations are needed to determine whether a significant prevention could be achieved by more prolonged ketogenic diets and by testing this diet in less severe experimental models, and moreover, to analyze the diet effects on the later and chronic stages leading to epileptogenesis.

## Introduction

The temporal lobe epilepsy (TLE) is the most common form of focal epilepsy in humans, usually characterized by recurrent localized seizures in specific regions of the cerebral cortex, coupled with hippocampal atrophy and sclerosis^1–6^. Overall, it is reported that more than 30% of epileptic patients have drug-resistant seizures^4,7^.

The ketogenic diet (KD) is a high-fat, low-carbohydrate diet already used, for decades, as an alternative therapy in refractory TLE^8,9^. In clinical trials, it has been proven to be effective in reducing seizures by 50% after 3 months of administration compared to the control groups, with response maintained for up to one year. Therefore, KD remains an attractive option especially, in children and adolescents with refractory epilepsy for whom surgery was ruled out^9–11^. Recently, international experts recommend the initiation of the KD within the first week after the diagnosis during the acute phase, with the need to be continued for up to 3 months (post-acute phase) if proven to be effective^12^.

In animal studies, the effect of ketogenic diet on epilepsy treatment has been already studied, often involving its administration before inducing a status epilepticus^13–17^. Neurons and astrocytes exhibit the capacity to absorb and catabolize β-hydroxybutyrate and acetoacetate, using ketone bodies in mitochondria for energy production and amino acid synthesis^18–20^.

In vivo imaging techniques, such as [^18^F]-fluorodeoxyglucose positron emission tomography ([^18^F]-FDG PET) and magnetic resonance imaging (MRI) can be used as two complementary noninvasive imaging techniques to evaluate brain metabolic changes, particularly the glucose metabolism, at an early stage of epilepsy^4,21^. Their combination provides functional information from molecular PET imaging and morphological information from the MRI. Their coupling with quantitative analyses brings new diagnostic solutions by giving complementary elements on the detection of small brain abnormalities and on the characterization of the seizure location^4,21–23^. In a previous study from our team, the changes in the brain glycolytic metabolism have been characterized and quantified using [^18^F]-fluorodeoxyglucose positron emission tomography (PET) imaging in a lithium-pilocarpine epilepsy model, displaying an early hypermetabolism on site known to be involved in the epileptogenesis process (piriform and entorhinal cortex, hippocampus)^21^.

One of the most recognized method for semi-quantitative analysis of neuroimaging data is Statistical Parametric Mapping (SPM), widely used in preclinical studies^21,24^. SPM also tends to improve the visual assessment in various clinical studies^25–28^. It improves reproducibility and is a time saver compared to visual analyses.

By using [^18^F]-FDG PET and a dedicated voxel-based quantification pipeline, the present study aimed to assess the effect of a short-term ketogenic diet (7 days) on cerebral metabolic changes, before and after the status epilepticus (SE) and especially within the brain areas known to be involved in the later stages of epiletogenesis development.

## Results

### Ketogenic diet before SE

For the 15 KD-rats at D-1, the 7-days of the KD were associated with significant increases of the body weight (321 ± 25 vs. 256 ± 23 g, *p* < 0.05) and of the plasma β-hydroxybutyrate (1.67 ± 0.37 vs.1.16 ± 0.36 mmol/L, *p* < 0.05), but with a stable glycemia (6.11 ± 0.85 vs. 6.59 ± 1.11 mmol/L, NS) (Table 1).

**Table 1:** Effect of the diet treatment on the physiological parameters.

	Baseline	7-days of KD	*P* values
Weight (g)	256 ± 23	321 ± 25	*p* < 0.01
Ketone bodies (mmol/L)	1.16 ± 0.36	1.67 ± 0.37	*p* < 0.05
Glycemia (mmol/L)	6.11 ± 0.85	6.59 ± 1.11	NS

**Figure 1:**
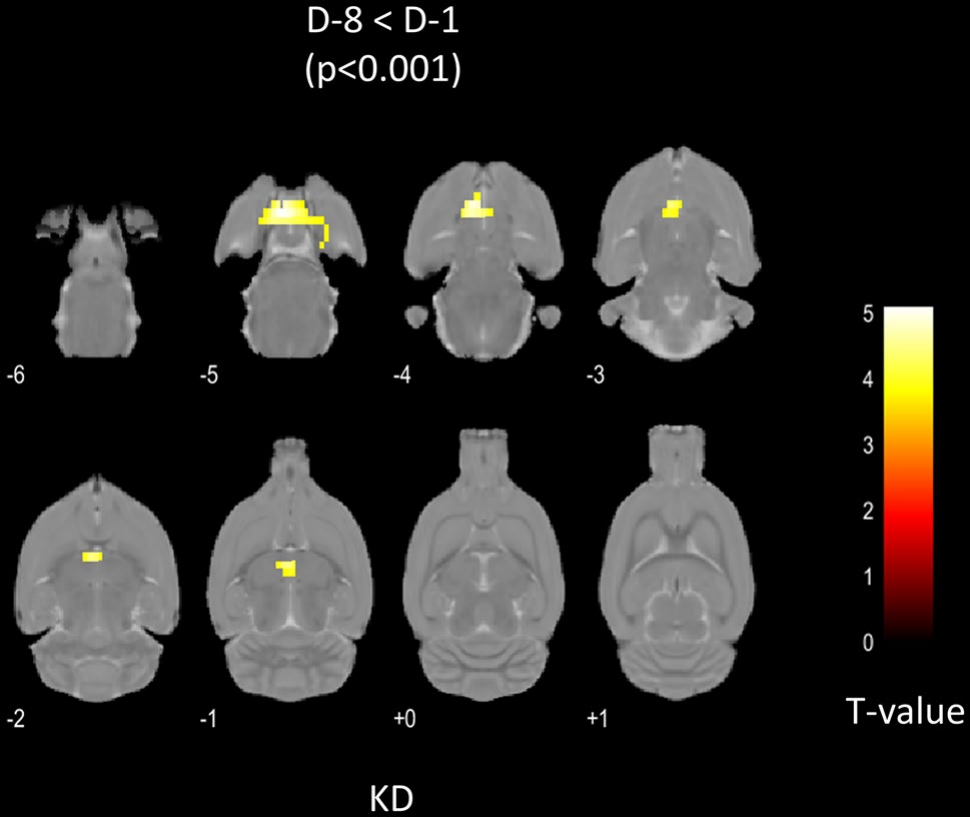
Study design. D-8 and D-1 are defined as eight and one day before status epilepticus (SE) respectively, H+4 is defined as four hours after SE.

The paired comparisons of the PET images before (D-8) and after (D-1) the KD showed areas of significant enhanced glycolytic brain metabolism at D-1, mainly within the hypothalamus (increased metabolism volume = 21.54 mm3T-voxel max at 5.21) and the periaqueductal grey matter (9.88 mm3, T-voxel max at 5.76) In contrast, no hypometabolic areas at D-1 were observed. The details are reported in Fig. 1 and Table 2.

**Table 2:** Anatomical localization, volume and maximal T-scores of the areas of increased glycolytic metabolism observed with the semi-quantitative analysis in the 15 KD-rats after 7 days of KD (i.e., with a paired comparison between D-8 and D-1 PET images).

Anatomical location	Volume (mm^3^)	T-score max
L_Hypothalamic Region	11.4208	5.0997
R_Hypothalamic Region	10.1228	5.2107
L_Periaqueductal Gray	5.6511	5.7571
R_Periaqueductal Gray	4.2285	5.1876

Only the areas with a T-voxel > 3.79 and a cluster size > 38 voxels are reported.

### SE after the ketogenic or standard diets

On the day of the SE induction, the level of plasma β-hydroxybutyrate was higher for the KD-rats than for the SD-rats (1.67 ± 0.37 vs. 1.08 ± 0.3 mmol/L, *p* < 0.05), but there was no significant difference between these 2 groups, neither for the body weight (321 ± 25 vs. 358 ± 31 g, NS), nor for the glycemia (6.59 ± 1.11 vs. 6.72 ± 0.25 mmol/L, NS) (Table 3).

**Table 3:** Comparison of physiological parameters between the SD-rats and the KD-rats before the induction of the status epilepticus.

	SD-rats	KD-rats	*P* values
Weight (g)	358 ± 31	321 ± 25	NS
Ketone bodies (mmol/L)	1.08 ± 0.3	1.67 ± 0.37	*p* < 0.05
Glycemia (mmol/L)	6.72 ± 0.25	6.59 ± 1.11	NS

All pilocarpine-treated rats entered convulsive SE of which 4 SD rats died during or after 4 h of convulsive SE.

The characteristics of the lithium pilocarpine-induced SE state were not influenced by the ketogenic diet. In each group, the event characteristics were like those previously documented in this model^29–31^.The rats of all groups exhibited the same behavioral features after the lithium-pilocarpine administration. Within 5 min after the pilocarpine injection, the rats developed diarrhea, piloerection, and other signs of cholinergic stimulation. During the following 10–20 min, the rats exhibited head bobbing, scratching, chewing, and exploratory behavior. Recurrent seizures started after pilocarpine administration with associated episodes of head and bilateral forelimb myoclonus with the animals rearing up on their hind legs and falling. They progressed to SE around 30–40 min after the pilocarpine injection.

The recurrent seizure thresholds seemed to appear faster in KD rats compared to SD rats (12–30 min after the pilocarpine injection vs. 18 to 32 min, in the KD and SD rats respectively, *p* < 0.05). The SE severity, however, was similar between the two groups.

**Figure 2:**
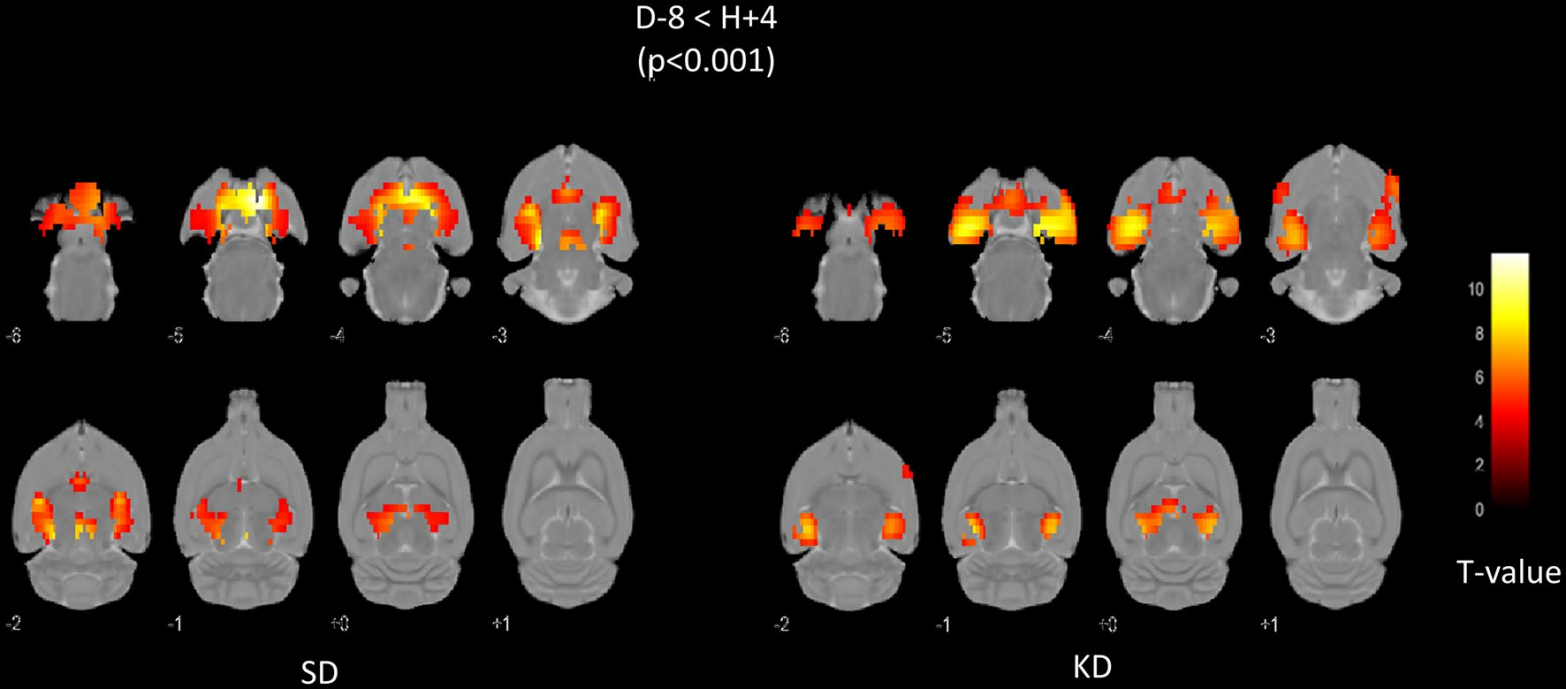
Representation of the spatial normalization pipeline. The PET images are normalized using non-linear transformations estimated between the SIGMA MRI (used to create the Atlas) and the individual MRI of each rat. PET images were previously registered to their individual MRI.

The results of the paired comparisons of PET images recorded before the SE (D-8) and at the 4th hour following the SE induction (H+4), are detailed in Fig. 2 for both groups and in Tables 4, 5 and Tables 6, 7 for the SD- and KD-rats’ groups, respectively. Both groups exhibited equivalent increases of the brain metabolism and within the same brain areas (Tables 4 and 6)., i.e., hippocampus (KD-rats: 71.83 mm^3^, T-voxel max at 15.52 vs. SD-rats: 68.77 mm^3^, 15.48), parahippocampic areas (83.20 mm^3^*,* 8.10 vs. 45.29 mm^3^, 11.05), thalamic and hypothalamic areas (20.77 mm^3^*,* 5.37 vs. 50.22 mm^3^, 11.62), periaqueductal gray (4.10 mm^3^, 5.62 vs. 17.85 mm^3^, 10.12), limbic structures (33.48 mm^3^, 8.10 vs. 4.26 mm^3^, 5.53). An increase of the brain metabolism was observed on the motor cortex for KD-rats (32.57 mm^3^, 8.23). Hypometabolic areas were also observed in some areas as in the corpus callosum or in the cingular cortex (Table 5 and 7).

**Table 4:** Anatomical localization, volume and maximal T-scores of the increased glycolytic metabolism areas observed with the semi-quantitative analysis in the 15 KD-rats during SE (i.e., with a paired comparison between the PET images recorded before SE (D-8) and at the 4th hour from SE induction (H+4)).

Anatomical location	Volume (mm^3^)	T-score max
L_Amygdalopiriform Cortex	7.4059	7.9502
L_Primary Cingular Cortex	5.861	6.917
L_Cornu Ammonis 1 (CA-1)	11.9768	7.8038
L_Cornu Ammonis 3 (CA-3)	8.8161	7.2452
L_Dentate Gyrus (DG)	13.7729	7.8038
L_Subiculum	7.9662	7.9502
L_Posterior Agralunar Insular Cortex	4.1771	6.8693
L_Primary Motor Cortex	11.8581	8.2289
L_Secondary Motor Cortex	5.2275	7.2986
L_Entorhinal Cortex	22.7396	7.9502
L_Lateral Entorhinal Cortex	8.3694	7.8838
L_Primary Somatosensory Cortex Barrel field	5.082	6.4075
L_Primary Somatosensory Cortex Dysgranular	5.0972	7.3051
L_Primary Somatosensory Cortex Forelimb	7.4037	8.0512
L_Ectorhinal Cortex	5.1559	6.1765
L_Hypothalamic Region	11.5852	5.3682
L_Olfactory Bulb	4.1844	4.5033
L_Corpus Callosum and Associated Subcortical White Matter	5.4483	6.2697
R_Amygdalopiriform Cortex	5.4433	8.0963
R_Primary Cingular Cortex	6.4061	7.0226
R_Cornu Ammonis 1 (CA-1)	8.3673	8.0963
R_Cornu Ammonis 3 (CA-3)	7.9264	7.6248
R_Dentate Gyrus (DG)	15.5198	7.6248
R_Subiculum	7.9018	8.0963
R_Primary Motor Cortex	10.2864	6.7354
R_Secondary Motor Cortex	5.2015	7.0226
R_Entorhinal Cortex	20.1515	8.0963
R_Lateral Entorhinal Cortex	5.9392	7.7323
R_Perirhinal Area 36	4.9691	7.3894
R_Primary Somatosensory Cortex Barrel field	4.6455	5.0633
R_Primary Somatosensory Cortex Forelimb	6.3924	5.3638
R_Hypothalamic Region	9.1853	5.2842
R_Periaqueductal Gray	4.0953	5.6234
R_Descending Corticofugal Pathways and Globus Pallidum (GP)	5.3021	6.8814

Only the areas with a T-voxel > 3.79 and a cluster size > 4 mm^3^ are reported.

**Table 5:** Anatomical localization, volume and maximal T-scores of the decreased glycolytic metabolism areas observed with the semi-quantitative analysis in the 15 KD-rats during SE (i.e., with a paired comparison between the PET images recorded before SE (D-8) and at the 4^th^ hour from SE induction (H+4)).

Anatomical location	Volume (mm^3^)	T-score max
L_Corpus Callosum and Associated Subcortical White Matter	4.1525	8.1072
R_Secondary Cingular Cortex	4.4167	9.8003
R_Corpus Callosum and Associated Subcortical White Matter	9.9939	10.3459

Only the areas with a T-voxel > 3.79 and a cluster size > 4 mm^3^ are reported.

**Table 6:** Anatomical localization, volume and maximal T-scores of the areas of increased glycolytic metabolism observed with the semi-quantitative analysis in the 11 SD-rats after SE (i.e., with a paired comparison between the PET images recorded before SE (D-8) and at the 4th hour after SE induction (H+4)).

Anatomical location	Volume (mm^3^)	T-score max
L_Amygdalopiriform Cortex	4.2604	5.53
L_Cornu Ammonis 1 (CA-1)	8.7828	9.0226
L_Cornu Ammonis 3 (CA-3)	10.8381	10.7581
L_Dentate Gyrus (DG)	16.7787	10.7581
L_Subiculum	6.8434	7.8296
L_Entorhinal Cortex	19.4978	11.0467
L_Hypothalamic Region	15.3923	11.6162
L_Periaqueductal Gray	8.958	8.4183
L_Thalamus	8.7386	9.6197
L_Descending Corticofugal Pathways and Globus Pallidum	10.2589	10.7581
R_Cornu Ammonis 1 (CA-1)	6.3373	5.6954
R_Cornu Ammonis 3 (CA-3)	10.5564	7.5118
R_Dentate Gyrus (DG)	15.4792	10.1283
R_Subiculum	5.4722	6.2329
R_Entorhinal Cortex	13.4754	8.4688
R_Hypothalamic Region	15.2077	9.0006
R_Periaqueductal Gray	8.8892	10.1228
R_Thalamus	10.8793	8.8559
R_Descending Corticofugal Pathways and GP	9.9606	10.1283

Only the areas with a T-voxel > 3.79 and a cluster size > 4 mm^3^ are reported.

**Table 7:** Anatomical localization, volume and maximal T-scores of the decreased glycolytic metabolism areas observed with the semi-quantitative analysis in the 11 SD-rats during SE (i.e., with a paired comparison between the PET images recorded before SE (D-8) and at the 4th hour from SE induction (H+4)).

Anatomical location	Volume (mm^3^)	T-score max
L_Primary Cingular Cortex	8.9153	13.102
L_Secondary Cingular Cortex	4.8793	14.3603
L_PreLimbic System	6.931	11.5046
L_Primary Motor Cortex	4.4645	8.7429
L_Corpus Callosum and Associated Subcortical White Matter	10.4305	13.9271
R_Primary Cingular Cortex	4.7657	12.3125
R_Secondary Cingular Cortex	4.5905	12.6772
R_Corpus Callosum and Associated Subcortical White Matter	5.2913	12.1243

Only the areas with a T-voxel > 3.79 and a cluster size > 4 mm^3^ are reported.

No significant differences were observed for the unpaired comparisons between the [^18^F]-FDG PET images from SD- and KD-rats at the 4th hour following SE induction.

## Discussion

After status epilepticus (SE), a dynamic process start, leading to an abnormal brain network reorganization^32^, metabolic changes and neurodegeneration, contributing to the establishment of the chronic disease. On the rat lithium-pilocarpine model, a brain glycolytic hypermetabolism occurring 1 to 4 h after SE, has already been documented in the areas known to be involved in the subsequent epileptogenesis process (mostly in the regions related to SE propagation as piriform cortex, amygdala, CA1 of the hippocampus, and the hilus of dentate gyrus (DG))^21,29,30^.

Therefore, by using [^18^F]-FDG-PET and a dedicated voxel-based quantification pipeline, we attempted to investigate the impact of a short-term ketogenic diet (7 days) on the brain glycolytic metabolism, in a well-known and validated model of TLE, the rat lithium-pilocarpine model, with a special focus on the metabolism of the brain areas known to be involved in the subsequent epileptogenesis process.

To reduce the variation in glucose metabolism caused by a KD, we tested the effects of this diet on the brain metabolism in healthy rats. The diet was well tolerated, with the animals considerably gaining weight within one week. The blood level of the ketone bodies was also significantly increased, confirming previous works reporting a drastic carbohydrate reduction during 3 to 5 days, can induce a ketosis^15,33^. However, we noted that glycemia remained unchanged despite the 7-day low-carbohydrate diet in accordance with results from previously published studies^34–36^.

We used a voxel-based method for the PET images analysis (SPM), which provides an objective semi-quantification of the brain metabolic changes. We specifically designed a dedicated SPM pipeline for this study. The spatial normalization step using anatomical MRI images guarantees the best localization and ensures a total independence of the normalization and statistical analyses^37^. It is not the case when the voxel information from the PET scans is used in the two steps. Actually, it might lead to a loss of spatial accuracy and to a dispersion of the voxel values and then, to a loss of power of the statistical analysis^38,39^. This statement is particularly relevant in our study for H+4 acquired images. In fact, the important hypermetabolism during SE is responsible for large differences between the to-be-normalized images and the PET template. The intensity normalization is also a crucial step in [^18^F]-FDG semi-quantitative studies. The concentration reaching the brain is subject-dependent because of different physiological factors, such as, among others, the blood glucose level, medications, and age^40^. The proportional scaling is widely used but was not adapted for our study. In fact, a significantly higher global intensity at H+4 (in comparison with D-8) can lead to an attenuation of the hypermetabolism areas and create an artificial hypometabolism in normal areas on H+4 normalized images. In previous studies on the lithium-pilocarpine rodent model^21,41,42^, the pons was recommended as the reference region for the intensity normalization, but we did not use it in our study in order to take in consideration the metabolism variations within the pons due to the ketogenic diet. Opposed to the anatomical reference region methods, the histogram-based method used in this paper is data-driven and efficient to detect abnormal patterns without requiring previous knowledge about the analyzed model or disease^43^. This method has already been used in other preclinical studies^44^ and also proved its efficiency in a clinical context^40^. Indeed, although they are present, the artificial hypometabolic areas at H+4 (vs D-8) are limited and considered as false positive because no metabolism decrease during SE is reported in the literature.

This pipeline was firstly applied to the [^18^F]-FDG PET images recorded in healthy rats before (D-8) and after a 7-days (D-1) ketogenic diet (KD) before the induction of the epilepsy. Paired analyses gave clear evidence of a KD-related hypermetabolism in periaqueductal gray (PAG) and hypothalamic areas. In the brain, these structures are activated through the food intake and reward processes and have extensive connections mediating the appetitive and the consummatory behaviors^45^. The periaqueductal gray matter has long been known as a region modulating fear and anxiety-related reactions^46–49^. More recently, its role in regulating and driving appetitive behaviors and its connections to the central feeding network of the brain, such as the hypothalamus, have been explored^48,50^. Likewise, previous study has also shown that the inactivation of PAG in rats alters the hypothalamic signaling and the food consumption^45^. Our data provide new evidence that hypothalamic areas and PAG may play a major role in appetitive behaviors and that 7 days of ketogenic diet can induce brain metabolic changes in normal rats. In contrast, we observed no decrease in brain metabolism after a 7-day KD, showing a physiological consumption of glucose in other areas of the brain. This is in accordance with the observation of an unchanged plasma glucose in our KD rats. It must be kept in mind that complex metabolic adaptations may influence glucose regulation during short KD periods. More precisely, it is likely that certain enzymes of glucose metabolism have not yet been significantly downregulated after a 7-day period (i.e., metabolic adjustments could take a longer time to stabilize)^51^. In addition, it has been postulated that the control of metabolic enzymes and transporters could vary between different brain areas^52^. Finally, as suggested by Stincone et al., the acute use of ketone bodies during KD could lead to a paradoxical increase in the glucose release within blood, in order to maintain important functions such as the cytoplasmic antioxidant defense^51,52^.

After the SE induction, the SPM analysis showed a comparable increased metabolism within the primary sites that are subsequently implicated in the epileptogenesis process (hippocampic and parahippocampic areas, entorhinal and piriform cortices and thalamic and hypothalamic areas)^15,21,30^ in the KD group and in the SD group at H+4 compared to D-8. We observed slightly more pronounced hypermetabolic volumes in the KD group compared to the SD group. Several hypermetabolism areas, were also detected in the KD but not in the SD group, such as motor cortex (Tables 4 and 5). However, the inter-group comparisons do not show a significant difference in brain [^18^F]-FDG uptake, at H+4 of the SE, between the KD and SD rats using SPM.

The inability of the KD to prevent lithium-pilocarpine SE was already documented. A prior investigation by Linard et al. explored the effects of the ketogenic diet in the lithium-pilocarpine rat model, using an electroencephalogram and histological analysis. Their study demonstrated a significant decrease in hippocampal neuronal loss, specifically in CA1 and CA3 regions, in the KD-rats compared to SD-rats. However, no significant difference were observed in the characteristics of clinical events during the acute phase of lithium-pilocarpine SE between the two groups^15^.

### Limitations and perspectives

Although the lithium-pilocarpine model reproduces the main clinical features of human TLE, extrapolation to the human epileptogenesis process remains difficult. In addition, it is likely that the KD effects could be different between rats and humans.

No histopathologic analyses were performed in the present study, which constitutes an additional limit. However, a number of previous studies published on this model, including a study from our team, have shown that most damaged neurons were located in the structures showing a significant increase in the metabolic activity during the status epilepticus^30,53–56^. The neuron damages and their potential prevention by a diet would be closely tied to the capacity of neurons to manage the extreme metabolic demands (3 to 6-folds increases) from certain brain regions^57,58^.

In these conditions and despite the preference for ketones, these brain areas should maintain a high glycolytic activity to meet the energy needs of the lithium-pilocarpine model.

As already discussed above, a 7-day KD may not be sufficiently long to complete the transition of the brain into a state of complete ketosis, using ketone bodies as the exclusive energy substrate. Achieving all the requisite metabolic alterations in the brain, might require a more prolonged period of ketogenic intervention.^59,60^.

The acute SE produced by the lithium-pilocarpine model is particularly aggressive, and it could be interesting to test KD on model gradually developing clinical and electroencephalographic seizure manifestations, such as the pentylenetetrazol (PTZ) perfusion rat model.

Another limitation is that the glycolytic metabolism assessed with [^18^F]-FDG-PET would not precisely correspond to the severity of neuronal loss^61^. The area of abnormal metabolism identified by interictal [^18^F]-FDG-PET often extends well beyond the epileptogenic zone, covering a large portion of the temporal lobe and surrounding regions^55,61^. Consequently, it may be worthy to explore alternative radiotracers such as [^18^F]flumazenil for a more precise delineation of the epileptogenic areas^62,63^.

A last limitation is that our study did assess the early stage of SE but did not extend to the later and chronic stages of the epileptogenesis process. A continued administration of throughout KD the chronic phase of epilepsy, would have provided a more comprehensive understanding of the KD impact on the epileptogenic process.

## Conclusion

A one-week ketogenic diet does not prevent the status epilepticus (SE) and metabolic brain abnormalities in the lithium-pilocarpine rat model. Further explorations are needed to determine whether a significant prevention could be achieved by more prolonged ketogenic diets and by testing this diet in less severe experimental models, and moreover, to analyze the diet effects on the later and chronic stages leading to epileptogenesis.

## Methods

### Animals and study design

All experiments involving animals complied with the ARRIVE guidelines and were approved by the Lorraine Ethics Committee N°66 according to Guidelines of Animal Care and Use (*APAFIS no. *16424-2018080814271998). Sprague–Dawley male rats (175–200 g) were purchased from Janvier Laboratories (Le Genest-Saint-Isle. France) and housed in ventilated cages including filter tops, under controlled environmental conditions (22 ± 2 °C, 55 ± 20% humidity) and 12-h light‐dark cycle.

**Figure 3:**
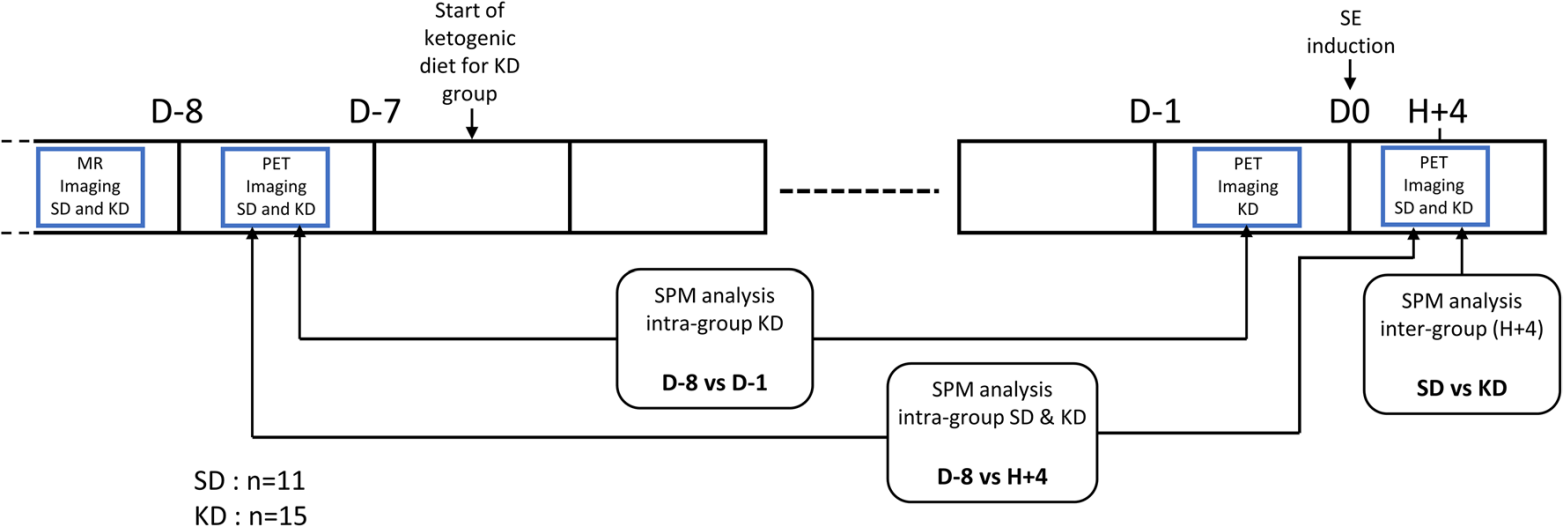
Anatomical localization of the areas of increased metabolic activity between Day (-8) and Day (-1) and for KD subjects (n = 11). The SPM-T maps were obtained using a paired test (*p* < 0.001, uncorrected, k > 50 voxels) then projected onto two-dimensional slices of T1-weighted MRI. The colorbar represents the T-values.

The imaging timeline is illustrated in Fig. 3. A brain [^18^F]-FDG PET was recorded in 24 adult male rats (D-8) and then, after 7 days of a KD (D-1, Fig. 3) (3% carbohydrate, 73% fat, 15% protein, 0% fiber, 9% vitamins, and minerals; ketocal^®^ SDS. France)^31,64,65^. A status epilepticus (SE) was thereafter induced in these KD-rats, with a third [^18^F]-FDG PET being recorded 4 h after the SE induction (H+4). A total of 24 KD rats were initially included in the study, but 4 rats had to be withdrawn due to technical issues encountered during the PET experiments. Additionally, 5 other rats were excluded from the analysis due to either a lack of MRI scans or the poor quality of PET images.

The same protocols of SE induction and H+4 [^18^F]-FDG PET recording were applied, for comparisons, in a control group of 15 rats submitted to a standard diet (SD-rats; 44% carbohydrate, 6% fat, 19% protein, 18% fiber, 13% vitamins, and minerals; Envigo. Gannat. France). In these SD-rats, a baseline [^18^F]-FDG PET scan was also performed, 7 days before SE induction (D-8). Moreover, for both groups, an MRI was realized before D-8. Food and water were given ad libitum in both groups and each rat was weighed daily at a fixed time with a dedicated small-animal scale (Mettler Toledo. DeltaRange-PR5002. France). Before each PET, two drops of blood were taken from the tip of the tail for the measurement of blood glucose and β-hydroxybutyrate levels by using Medisense Optium Xceed reader (Abbott France Division Medisense. Rungis.France)^64^.

### Induction of the Status epilepticus

The SE was induced as described in previous studies^21,64^, by a subcutaneous injection of pilocarpine at a dose of 25 mg/kg, 1 h after a subcutaneous injection of 1 mg/kg of methyl scopolamine which limits the peripheral effects of pilocarpine, and 20 h after an intraperitoneal injection of 3 mEq/kg of lithium chloride allowing a significant reduction of the dose of pilocarpine required for seizure induction. Two hours after the onset of SE, all rats received an intramuscular injection of diazepam for muscle relaxation without halting status epilepticus (2.5 mg/kg, i.m. Valium; Roche.Basel, Switzerland)^21,64^. To assess the occurrence of seizures, all rats were monitored and video-recorded, for 4 h, immediately after pilocarpine injection. The SE was considered to start after 5 seizures including these three consecutive stages: clonic seizures, rearing and falling^31,53,64^.

### PET imaging

Using a previously described methodology^21^, approximately 0.2 MBq/g of [^18^F]-FDG were injected in the tail vein under short-term anesthesia by inhalation of an isoflurane-oxygen mixture (2%–1.5 v/v) in the SD- and KD-rats. Then, the rats were put in their home cage back, in a quiet environment. The brain [^18^F] FDG-PET was recorded 45 min later with an Inveon PET system (Siemens. Knoxville. TN. USA), under the same isoflurane-oxygen anesthesia and with a 30-min emission sequence followed by a 10-min transmission sequence with Co-57 providing an attenuation correction map. Their respiration was monitored and maintained constant throughout the experiment. As previously described^21^, the brain [^18^F]-FDG PET images were reconstructed in kBq/ml using an OSEM-3D iterative method involving 4 iterations with 12 subsets and corrected for attenuation. The images were finally displayed with 0.26 × 0.26 × 0.80 mm^3^ voxels, reoriented and cropped to suppress most of the extracerebral signal.

### MR imaging

Animals were anesthetized by inhalation of an isoflurane-oxygen mixture (3%–1.5 v/v) and their respiration was monitored and maintained constant throughout the experiment. Anatomical MRI reference images of the brain were obtained on a 3 Tesla scanner (Prisma, Siemens Healthineers^®^, Erlangen, Germany) with a rat-dedicated 8-channel volume coil (Rapid Biomedical GmbH^®^, Rimpar, Germany). T2‐weighted (T2-w) anatomical images were acquired using a 2D Turbo Spin-Echo sequence and with the following parameters: repetition time (TR)/echo-time (TE) = 2500/61 ms, voxel size = 0.255 × 0.255 × 1 mm3, 24 slices from the olfactory bulb to the brain stem, field of view (FOV) = 49 × 49 mm^2^, 8 averages.

At the end of the study, animals were sacrificed by intraperitoneal injection of Euthasol overdose (200 mg kg^−1^; Euthasol Vet. 400 mg ml^−1^).

### Statistical parametric mapping

The PET images were pre-processed using SPM12 (Wellcome Department of Cognitive Neurology, Institute of Neurology, London, UK) running on Matlab 2020a (MathWorks Inc., Sherborn, MA).

**Figure 4:**
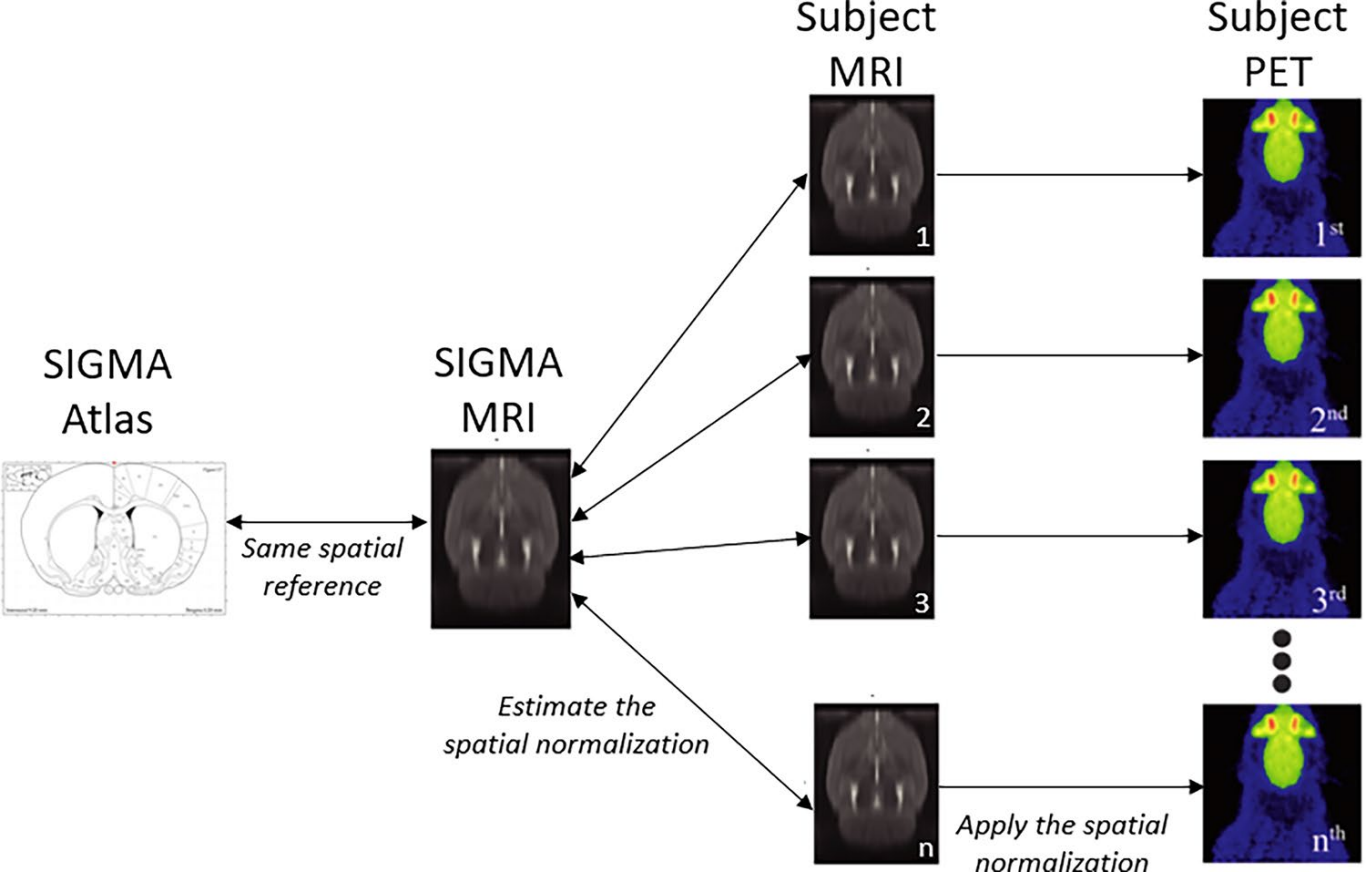
Areas of increased [^18^F]-FDG uptake observed at the 4th hour after induction of SE (i.e., with a paired comparison between the PET images recorded before SE and at the 4^th^ hours after SE induction) in the 11 SD-rats (left panel) and 15 KD-rats (right panel). SPM-T maps are displayed with a Z score-based color scale, projected on T1-weighted MRI slices, and with the following parameters: *p* < 0.001 (no correction for multiple comparisons), and k > 38 voxels. The colorbar represents the T-values.

MRI images of each animal were spatially normalized using the SIGMA MRI rat brain template^66^. The corresponding non-linear transformations were applied to the PET images previously co-registered to the MRI images of the same rats (see Fig. 4 for the overall normalization pipeline), and an isotropic 3D Gaussian kernel of 0.8 mm FWHM was subsequently applied. An intensity normalization was performed using the histogram-based (HB) method of Fuster et al.^43^ with a control PET template obtained by averaging the spatially normalized PET images of the 11 control rats (before the SE induction, D-8).

Paired sample t-tests were used to assess the brain metabolic effects of (i) the ketogenic diet before the SE induction, while comparing the D-8 and D-1 normalized PET images of KD group, and (ii) the SE while comparing the D-8 and H+4 normalized PET images of both KD and SD groups. Unpaired two-sample t-tests were also applied for direct comparisons of the KD and SD groups at H+4. For all tests and for each voxel setting in the main sites known to be involved in the epileptogenesis process of this model, notably the temporal cortical area (entorhinal and piriform cortex), the hippocampal and parahippocampic areas^41,67,68^, a *p* value < 0.001 corrected for cluster volume (by using the expected volume provided by SPM and based on the random field theory) was considered to reflect a significant metabolism change. Others regions were masked using the SIGMA rat brain atlas^66^.

### Statistical analysis

All data are expressed as mean ± SEM. Statistical analyses were performed using the SPSS Statistics Software package v. 20 (IBM, NY, USA). Comparisons of quantitative variables were performed with ANOVA-test after verifying for distribution normality. *P* values < 0.05 were considered as statistically significant.

### Ethical approval

All applicable international. national. and/or institutional guidelines for the care and use of animals were followed. This study was approved by the Lorraine Ethics Committee on Animal Experimentation (CELMEA LORRAIN N°66). (APAFIS number. 16424-2018080814271998).

## Data Availability

The datasets generated during the current study are available from the corresponding author on reasonable request.

## References

[1] Barañano KW, Hartman AL (2008). The ketogenic diet: Uses in epilepsy and other neurologic illnesses. Curr. Treat Options Neurol..

[2] Simeone TA, Simeone KA, Stafstrom CE, Rho JM (2018). Do ketone bodies mediate the anti-seizure effects of the ketogenic diet?. Neuropharmacology.

[3] French JA (1993). Characteristics of medial temporal lobe epilepsy: I. Results of history and physical examination. Ann. Neurol..

[4] Verger A (2016). Temporal epilepsy lesions may be detected by the voxel-based quantitative analysis of brain FDG-PET images using an original block-matching normalization software. Ann. Nucl. Med..

[5] Ren E, Curia G (2021). Synaptic reshaping and neuronal outcomes in the temporal lobe epilepsy. Int. J. Mol. Sci..

[6] Twible C, Abdo R, Zhang Q (2021). Astrocyte role in temporal lobe epilepsy and development of mossy fiber sprouting. Front. Cell Neurosci..

[7] Weaver DF, Pohlmann-Eden B (2013). Pharmacoresistant epilepsy: unmet needs in solving the puzzle(s). Epilepsia.

[8] Peterman MG (1925). The ketogenic diet in epilepsy. J. Am. Med. Assoc..

[9] Neal EG (2008). The ketogenic diet for the treatment of childhood epilepsy: A randomised controlled trial. Lancet Neurol..

[10] Lambrechts DJE (2017). A randomized controlled trial of the ketogenic diet in refractory childhood epilepsy. Acta Neurol. Scand..

[11] Sourbron J (2020). Ketogenic diet for the treatment of pediatric epilepsy: review and meta-analysis. Childs Nerv. Syst..

[12] Wickstrom R (2022). International consensus recommendations for management of New Onset Refractory Status Epilepticus (NORSE) including Febrile Infection-Related Epilepsy Syndrome (FIRES): Summary and Clinical Tools. Epilepsia.

[13] Bough KJ (2006). Mitochondrial biogenesis in the anticonvulsant mechanism of the ketogenic diet. Ann. Neurol..

[14] Bough KJ, Valiyil R, Han FT, Eagles DA (1999). Seizure resistance is dependent upon age and calorie restriction in rats fed a ketogenic diet. Epilepsy Res..

[15] Linard B, Ferrandon A, Koning E, Nehlig A, Raffo E (2010). Ketogenic diet exhibits neuroprotective effects in hippocampus but fails to prevent epileptogenesis in the lithium-pilocarpine model of mesial temporal lobe epilepsy in adult rats. Epilepsia.

[16] Appleton DB, DeVivo DC (1974). An animal model for the ketogenic diet. Epilepsia.

[17] Rho JM, Szot P, Tempel BL, Schwartzkroin PA (1999). Developmental seizure susceptibility of kv11 potassium channel knockout mice. Dev. Neurosci..

[18] Nehlig A (1999). Age-dependent pathways of brain energy metabolism: the suckling rat, a natural model of the ketogenic diet. Epilepsy Res..

[19] Sokoloff L (1977). The [14 C]Deoxyglucose method for the measurement of local cerebral glucose utilization: theory, procedure, and normal values in the conscious and anesthetized albino rat. J. Neurochem..

[20] Martinez-Hernández A, Cloche K, Norenberg M (1977). Glutamine synthétase : localisation glial. Glutamine synthétase Localisation gliale dans le cerveau. Sciences.

[21] Poussier S (2017). Quantitative SPM analysis involving an adaptive template may be easily applied to [18F]FDG PET images of the rat brain. Mol. Imaging Biol..

[22] Willmann O, Wennberg R, May T, Woermann FG, Pohlmann-Eden B (2007). The contribution of 18F-FDG PET in preoperative epilepsy surgery evaluation for patients with temporal lobe epilepsy A meta-analysis. Seizure.

[23] Prieto E (2011). Statistical parametric maps of ^18^F-FDG PET and 3-D autoradiography in the rat brain: a cross-validation study. Eur. J. Nucl. Med. Mol. Imaging.

[24] Zhang L (2015). FDG-PET and NeuN-GFAP immunohistochemistry of hippocampus at different phases of the pilocarpine model of temporal lobe epilepsy. Int. J. Med. Sci..

[25] Kim YK (2002). (18)F-FDG PET in localization of frontal lobe epilepsy: comparison of visual and SPM analysis. J. Nucl. Med..

[26] Kim MA (2006). Relationship between bilateral temporal hypometabolism and EEG findings for mesial temporal lobe epilepsy: analysis of 18F-FDG PET using SPM. Seizure.

[27] Plotkin M (2003). Use of statistical parametric mapping of (18) F-FDG-PET in frontal lobe epilepsy. Nuklearmedizin.

[28] Kumar A (2010). Objective detection of epileptic foci by 18F-FDG PET in children undergoing epilepsy surgery. J. Nucl. Med..

[29] Da Silva Fernandes MJ, Dubé C, Boyet S, Marescaux C, Nehlig A (1999). Correlation between hypermetabolism and neuronal damage during status epilepticus induced by lithium and pilocarpine in immature and adult rats. J. Cereb. Blood Flow Metab..

[30] Dubé C, Boyet S, Marescaux C, Nehlig A (2000). Progressive metabolic changes underlying the chronic reorganization of brain circuits during the silent phase of the lithium-pilocarpine model of epilepsy in the immature and adult Rat. Exp. Neurol..

[31] Raffo E, François J, Ferrandon A, Koning E, Nehlig A (2008). Calorie-restricted ketogenic diet increases thresholds to all patterns of pentylenetetrazol-induced seizures: Critical importance of electroclinical assessment. Epilepsia.

[32] Doyen M (2022). Metabolic connectivity is associated with seizure outcome in surgically treated temporal lobe epilepsies: A (18)F-FDG PET seed correlation analysis. Neuroimage Clin..

[33] Paoli A (2014). Ketogenic diet for obesity: Friend or foe?. IJERPH.

[34] Al-Mudallal AS, LaManna JC, Lust WD, Harik SI (1996). Diet-induced ketosis does not cause cerebral acidosis. Epilepsia.

[35] Melø TM, Nehlig A, Sonnewald U (2006). Neuronal–glial interactions in rats fed a ketogenic diet. Neurochem. Int..

[36] Kuter KZ, Olech Ł, Głowacka U, Paleczna M (2021). Increased beta-hydroxybutyrate level is not sufficient for the neuroprotective effect of long-term ketogenic diet in an animal model of early Parkinson’s disease. Exploration of brain and liver energy metabolism markers. IJMS.

[37] Gispert JD (2003). Influence of the normalization template on the outcome of statistical parametric mapping of PET scans. Neuroimage.

[38] Ashburner J, Friston KJ (1999). Nonlinear spatial normalization using basis functions. Hum. Brain Mapp..

[39] Martino ME (2013). Comparison of different methods of spatial normalization of FDG-PET brain images in the voxel-wise analysis of MCI patients and controls. Ann. Nucl. Med..

[40] López-González FJ (2020). Intensity normalization methods in brain FDG-PET quantification. NeuroImage.

[41] Goffin K, Van Paesschen W, Dupont P, Van Laere K (2009). Longitudinal microPET imaging of brain glucose metabolism in rat lithium–pilocarpine model of epilepsy. Exp. Neurol..

[42] Jupp B (2012). Hypometabolism precedes limbic atrophy and spontaneous recurrent seizures in a rat model of TLE. Epilepsia.

[43] Martí Fuster B (2013). FocusDET, a new toolbox for SISCOM analysis evaluation of the registration accuracy using Monte Carlo simulation. Neuroinform.

[44] Proesmans S (2021). Voxel-based analysis of [18F]-FDG brain PET in rats using data-driven normalization. Front. Med..

[45] Tryon VL, Mizumori SJY (2018). A novel role for the periaqueductal gray in consummatory behavior. Front. Behav. Neurosci..

[46] Rizvi TA, Ennis M, Behbehani MM, Shipley MT (1991). Connections between the central nucleus of the amygdala and the midbrain periaqueductal gray: topography and reciprocity. J. Comp. Neurol..

[47] Krukoff TL, Harris KH, Jhamandas JH (1993). Efferent projections from the parabrachial nucleus demonstrated with the anterograde tracer Phaseolus vulgaris leucoagglutinin. Brain Res. Bull..

[48] Krout KE, Jansen AS, Loewy AD (1998). Periaqueductal gray matter projection to the parabrachial nucleus in rat. J. Comp. Neurol..

[49] Depaulis A, Bandler R (2012). The midbrain periaqueductal gray matter: functional, anatomical, and neurochemical organization.

[50] Behbehani MM, Park MR, Clement ME (1988). Interactions between the lateral hypothalamus and the periaqueductal gray. J. Neurosci..

[51] Stincone A (2015). The return of metabolism: biochemistry and physiology of the pentose phosphate pathway. Biol. Rev..

[52] Zilberter Y, Zilberter M (2017). The vicious circle of hypometabolism in neurodegenerative diseases: Ways and mechanisms of metabolic correction. J. Neurosci. Res..

[53] Turski L, Ikonomidou C, Turski WA, Bortolotto ZA, Cavalheiro EA (1989). Review: Cholinergic mechanisms and epileptogenesis. The seizures induced by pilocarpine: A novel experimental model of intractable epilepsy. Synapse.

[54] Cavalheiro EA (1995). The pilocarpine model of epilepsy. Ital. J. Neuro Sci..

[55] Dubé C, Nehlig A (2001). Conséquences des crises épileptiques subintrantes sur le développement cérébral: Relation entre l’agression initiale et le développement d’une épilepsie temporale. Epilepsies.

[56] Perosa SR (2007). Kinin B1 and B2 receptors are overexpressed in the hippocampus of humans with temporal lobe epilepsy. Hippocampus.

[57] Maalouf M, Rho JM, Mattson MP (2009). The neuroprotective properties of calorie restriction, the ketogenic diet, and ketone bodies. Brain Res. Rev..

[58] Gasior M, Rogawski MA, Hartman AL (2006). Neuroprotective and disease-modifying effects of the ketogenic diet. Behav. Pharmacol..

[59] Kossoff EH (2018). Optimal clinical management of children receiving dietary therapies for epilepsy: Updated recommendations of the International Ketogenic Diet Study Group. Epilepsia Open.

[60] Stafstrom CE, Rho JM (2012). The ketogenic diet as a treatment paradigm for diverse neurological disorders. Front. Pharmacol..

[61] Foldvary. Correlation of Hippocampal Neuronal Density and FDG-PET in Mesial Temporal Lobe Epilepsy (1999). 10.1111/j.1528-1157.1999.tb01984.x 9924898

[62] Ryvlin P (1998). Clinical utility of flumazenil-PET versus [18F]fluorodeoxyglucose-PET and MRI in refractory partial epilepsy. A prospective study in 100 patients. Brain.

[63] Vivash L (2013). ^18^ F-Flumazenil: A γ-aminobutyric acid A-specific PET radiotracer for the localization of drug-resistant temporal lobe epilepsy. J. Nucl. Med..

[64] Linard B, Ferrandon A, Koning E, Nehlig A, Raffo E (2010). Ketogenic diet exhibits neuroprotective effects in hippocampus but fails to prevent epileptogenesis in the lithium–pilocarpine model of mesial temporal lobe epilepsy in adult rats. Epilepsia.

[65] Clément A (2020). A 1-week extension of a ketogenic diet provides a further decrease in myocardial 18F-FDG uptake and a high detectability of myocarditis with FDG-PET. J. Nucl. Cardiol..

[66] Barrière DA (2019). The SIGMA rat brain templates and atlases for multimodal MRI data analysis and visualization. Nat. Commun..

[67] Curia G, Longo D, Biagini G, Jones RSG, Avoli M (2008). The pilocarpine model of temporal lobe epilepsy. J. Neurosci. Methods.

[68] Guo Y (2009). In vivo mapping of temporospatial changes in glucose utilization in rat brain during epileptogenesis: An 18F-fluorodeoxyglucose-small animal positron emission tomography study. Neuroscience.

